# Prevalence of Sarcopenic Obesity and its Association with Functionality, Lifestyle, Biomarkers and Morbidities in Older Adults: the FIBRA-RJ Study of Frailty in Older Brazilian Adults

**DOI:** 10.6061/clinics/2020/e1814

**Published:** 2020-11-25

**Authors:** Glaucia Cristina de Campos, Roberto Alves Lourenço, Claudia S. Lopes

**Affiliations:** I Departamento de Epidemiologia, Instituto de Medicina Social, Universidade do Estado do Rio de Janeiro, RJ, BR; II Departamento de Medicina Interna - Faculdade de Ciencias Medicas, Universidade do Estado do Rio de Janeiro, RJ, BR

**Keywords:** Cross-Sectional Study, Elderly, Obesity, Sarcopenic Obesity

## Abstract

**OBJECTIVES:**

To assess the prevalence of sarcopenic obesity and its association with functionality, lifestyle, biomarkers, and morbidities in older adults.

**METHODS:**

The study analyzed cross-sectional data from 270 older adults who participated in phase III of the Frailty in Brazilian Older People Study (Fragilidade em Idosos Brasileiros—Rio de Janeiro, FIBRA-RJ study-2013). They took part in a home interview surveying socioeconomic, demographic, lifestyle, morbidities, and functional data. Blood was collected for biochemical marker analysis and participants’ body composition was determined by dual-energy X-ray absorptiometry. For women, the diagnosis of sarcopenic obesity was defined at a body fat percentage ≥38% and appendicular skeletal muscle mass index (ASMMI) <5.45 kg/m^2^. For men, a fat percentage ≥27% and ASMMI <7.26 kg/m^2^ was defined as sarcopenic obesity. Multivariate analysis was performed using a multinomial regression model (95% confidence intervals), with sarcopenic obesity as the outcome.

**RESULTS:**

The prevalence of sarcopenic obesity was 29.3%. In the final fitted model, the variables that displayed statistically significant association with sarcopenic obesity were lower gait speed, self-reported medical diagnosis of arthrosis or arthritis, and high levels of glycemia.

**CONCLUSION:**

The study showed a high prevalence of sarcopenic obesity in non-institutionalized older adults in Brazil. The finding that this condition was associated with modifiable risk factors may provide insights into measures directed at prevention and reduction of the risk of sarcopenic obesity in this population subgroup.

## INTRODUCTION

The diagnosis of sarcopenic obesity (SO) considers the simultaneous presentation of high fat mass and low muscle mass; it represents the confluence between two body-composition phenotypes: sarcopenia and obesity. In recent years, obesity and sarcopenia have come to be considered as global epidemics, having a substantial impact on older adults’ quality of life, mainly through the synergistic effects of these two factors.

Globally, and in Brazil, the prevalence of SO in older adults has increased in recent years (1-7). However, due to the use of different diagnostic criteria and methods, the reported prevalence has varied considerably.

Studies using anthropometric measurements and electrical bioimpedance to diagnose SO have tended to find prevalence values ranging from 2% to 25% (8-11). Studies using dual-energy X-ray absorptiometry (DXA) have observed higher prevalence values, ranging from 2.75% to 84% (2,3,12-19). Baumgartner (17) was one of the pioneers in defining SO using DXA as a diagnostic method (20,21).

The DXA assessment of body composition is less likely to be influenced by body water content than other methods. Measurements obtained by this method has been considered as a reference standard in validation studies of methods and equations used to assess body composition. This method offers good validity and reliability and is a non-invasive technique for collecting information on various human body tissue types (22-24).

Prior studies have found an association between SO and functionality, sedentarism, biomarkers such as C-reactive protein (CRP), glucose, and albumin, and diabetes mellitus (18,20,25,26). In a study of 2,264 older adults in Korea, Ryu et al. (18) showed that engaging in moderate physical activity was inversely associated with SO.

More recently, there has been increasing interest in elucidating the relationship between biomarkers and SO (26,27). In several studies, biochemical markers, such as C-reactive protein (CRP) (26), glucose, and albumin (25,28), have been associated with SO. The association was usually two-way, but there was some evidence that high CRP and insulin resistance can predict SO (26). A high CRP level is a non-specific clinical marker of inflammation, and is associated with muscle catabolism (20,26).

SO is associated with increased morbidity, mortality, reduced quality of life, increased hospital admissions, and higher health costs (7,29). However, the related studies have used different criteria and methods, and, in Brazil, few studies used DXA (1,30-32) as a method for evaluating SO, and none have investigated its associated factors in the present study.

Accordingly, the objective of this study was to evaluate the prevalence of SO in older adults in Brazil, and to investigate its association with functionality, lifestyle, particular biomarkers, and physical morbidity.

## MATERIAL AND METHODS

### Study design and population

The methodology of the Frailty in Brazilian Older Adults study (Fragilidade em Idosos Brasileiros, seção Rio de Janeiro, FIBRA-RJ), was described elsewhere (33). Briefly, this is a three-phase cohort study, whose sample population was sourced from the client database of a health care and social security foundation. In phase I, in 2009, the baseline sample was enrolled using the following inclusion criteria: being a client of the social security foundation for at least 1 year; being 65 years or older; and residing in one of the northern neighborhoods of Rio de Janeiro City, Brazil. The exclusion criteria were: having a Mini-Mental State Examination (MMSE) score of ≤13; having an active neuropsychiatric condition; having severe hearing or sight impairment that would prevent response to the questionnaire; having a limiting gait disturbance; or being a wheelchair user or bedbound. Participants were interviewed at their homes. The sampling plan was stratified by sex and age group. An inverse sampling strategy was applied to achieve a sample size representative of each stratum, making it unnecessary to increase the calculated sample size to offset the non-response rate (33). The first phase sample comprised 847 individuals.

The data analyzed in the present study were obtained from the phase III database recorded in 2013. From the phase I sample, 136 individuals were excluded due to death and 102 individuals for the following reasons: MMSE score of ≤13; institutionalization; wheelchair user or bedbound; pacemaker user; difficulty in walking; or severe cognitive changes reported by the family. After these exclusions, 609 individuals were eligible, and attempts were made to contact them to request participation. Of these, 64 could not be located, and 143 refused to take part. Thus, 402 older adults constituted the final sample; these individuals participated in home interviews, following the phase I protocol.

**Figure 1:**
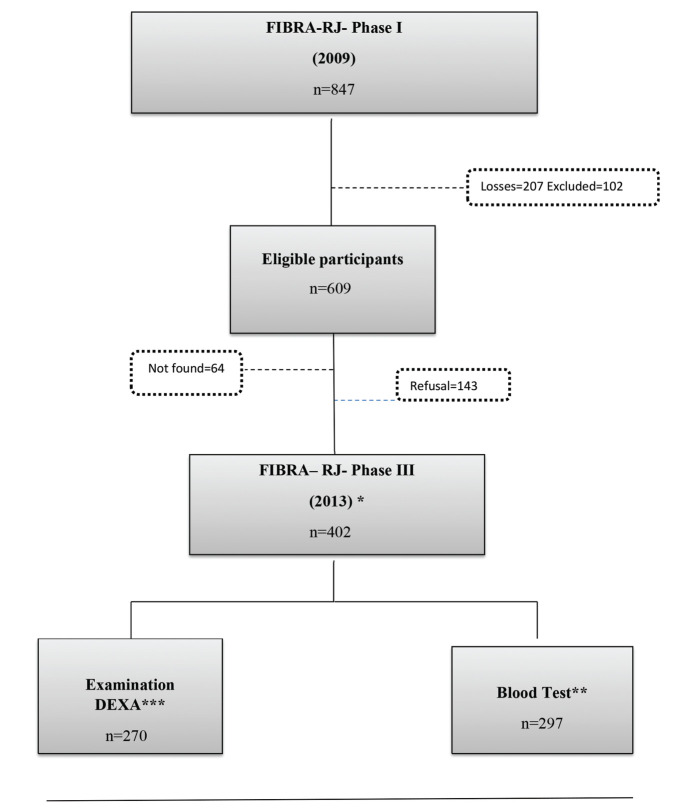
Diagram for selecting the final sample of the FIBRA RJ-III study. *GERONLAB researchers conducted a face-to-face interview with the participant at their residence and obtained anthropometric measurements. **Visit to the Human Aging Laboratory for blood tests. ***Visit of the elderly person to the Interdisciplinary Nutritional Assessment Laboratory to assess body composition through DEXA: Exclusion=4, not found=4, refusal=123, and deceased during the study=1.

For blood tests and body composition evaluation, all 402 individuals were invited to visit the clinical analysis and nutritional evaluation laboratories of Rio de Janeiro State University (Universidade do Estado do Rio de Janeiro, UERJ). Transport was offered to those with difficulty in traveling from home. Three hundred individuals agreed to proceed with the study; the blood test was performed on 297 and the DXA examination on 270 (Figure 1).

### Measurements and variables

#### Body composition

Weight and height were measured for all participants while barefoot and wearing light clothing, using the methods described by Lohman et al. (34). Height was measured using a tape measure fixed to the wall and was recorded in millimeters, with no rounding. Weight was measured using portable digital scales (Plenna/Everest TIN-00110), with a capacity of 150 kg and accurate to 200 g, and was recorded to a tenth of a kilogram, with no rounding.

DXA was used to evaluate body composition (General Electric, model GE Lunar IDXA). Fat mass and lean mass were analyzed. The upper and lower limbs were isolated from the trunk and head by lines drawn by the software, which could then be manually adjusted with precision. Appendicular muscle mass was evaluated by means of the appendicular skeletal muscle mass index (ASMMI), obtained by adding lean mass, *i.e.*, lean tissue (kg), from the arm and leg regions, and dividing by the square of the height (meters) (35).

#### Dependent variable

SO was diagnosed by a combination of variables used to diagnose obesity and sarcopenia as measured by DXA. Elderly individuals were classified as obese by body fat percentage cut-off values (≥38% for women and ≥27% for men) (17,36), and as sarcopenic when the ASMMI was <7.26 kg/m^2^ for men, and <5.45 kg/m2 for women (36). Accordingly, women with both a fat percentage ≥38% and ASMMI <5.45 kg/m2, and men with both a fat percentage ≥27% and ASMMI <7.26 kg/m2, were defined as having SO (17,36).

In order to evaluate the dependent variable, SO, participants were categorized as eutrophic, non-sarcopenic obese, or sarcopenic obese.

#### Independent variables

Factors potentially associated with SO were grouped into three groups: 1) functional variables and physical morbidities (gait speed, hand grip, instrumental activities of daily living, diabetes mellitus, and arthrosis/arthritis), 2) biomarkers (CRP, albumin, and glucose), and 3) lifestyle variables (tobacco use, daily consumption of alcohol, and regular walking for exercise).

#### Functional variables and physical morbidities

Gait speed was evaluated by measuring the time taken by the individual to walk 4.6 meters at their habitual pace. A chronometer was started at the moment the individual placed a foot on the starting mark and lifted the other off the ground. Individuals with gait speeds of 0.8 m/s or less were considered as displaying gait slowing (35).

Muscular strength was evaluated by taking three measurements at regular intervals of 1 minute using a hydraulic dynamometer (Jamar NC 701/42—North Coast, Rio de Janeiro, Brazil) in the dominant hand. The grip was adjusted according to the participant’s sex: position 2 for women and position 3 for men. The mean value of the three measurements was calculated and analyzed with adjustment for the individual’s sex and BMI. Individuals in the first performance quintile were considered to have low hand-grip strength (37).

Instrumental Activities of Daily Living (IADLs) were assessed using the Lawton scale (38), for seven day-to-day activities: using the telephone, shopping, leaving home alone, preparing meals, performing domestic chores, managing finances, and taking medicines. Individuals needing help in any one activity or more were considered dependent.

Physical morbidities—diabetes mellitus and arthrosis/arthritis—were investigated by self-reporting in answer to the question: “Has a doctor ever told you that you have the following health problems: diabetes mellitus (Yes/No) and arthrosis/arthritis (Yes/No)?”.

#### Biomarkers

CRP levels were considered abnormal at >0.5 mg/dl; albumin was considered low at <3.5 g/dl; and glycemia was considered high at >100 mg/dl. The participant’s blood samples were collected by trained technicians, by vacuum venipuncture, after a 12-hour fast. Participants abstained from physical exercise for 24 hours before collection, and from consuming alcohol in the 72 hours before collection.

#### Lifestyle

The lifestyle variables examined were tobacco use (current smoker/ever smoked and stopped/never smoked), walking for exercise (Yes/No), and consuming alcohol on a daily basis (Yes/No).

#### Covariables

The socioeconomic and demographic variables: age, sex, income, schooling, and race were included as covariables.

Age was recorded in years, in three age groups (65-74 years; 75-84 years; ≥85 years). Income source was described as retired, pensioners, and/or with other sources of income. Individual income from work, retirement benefit, or pension was recorded in minimum wages (MWs) at the time of interview and was categorized as 0-2 MWs; 2.1-5 MWs; and >5 MWs. Education was recorded in years and was categorized as illiterate, 1-5 years; 6-11 years, and ≥12 years. Race was categorized as white and Afro-descendent.

### Statistical analysis

For descriptive analysis, absolute and relative frequencies were calculated for the categorical variables. To evaluate the dependent variable, individuals were categorized as eutrophic, non-sarcopenic obese, and sarcopenic obese. A bivariate analysis was performed, using the chi-square test, to investigate the association between SO and the independent variables.

Subsequent multivariate analysis was performed by multinomial logistic model, which yielded crude and adjusted odds ratios (ORs) as measures of association, together with their respective 95% confidence intervals (95%CI). That model was used to identify associations of functional variables, morbidities, biochemical markers, and lifestyle with SO.

Independent variables that returned *p*<0.20 in the bivariate analysis were included in the multivariate models. We evaluate the fit of the model using the deviance statistic and the pseudo-R of Nagelkerke. Statistical analysis was conducted using the statistical package SPSS for Windows, version 19.

### Ethical considerations

The study followed the recommendations of Resolution 196/96 of the Ministry of Health’s National Health Council (Conselho Nacional de Saúde), and was approved by the National Research Ethics Committee (Comissão Nacional de Ética e Pesquisa, CONEP), in opinion No. 120.700/2012. All participants signed a declaration of free and informed consent.

## RESULTS

This study examined 270, predominantly female, older adults, of mean age 77.5 years (SD=5.92). The prevalence of SO was 29.3% (n=78). Most participants were white, with an income ranging from 2.1 to 5 MW, and were in the 75-84 year age group. Almost half had 6-11 years’ education, while about one-third had more than 12 years’ education (Table 1).

Table 2 shows the prevalence of SO, by socioeconomic, demographic, lifestyle, morbidity, biochemical marker, and functional characteristics. The prevalence of SO was markedly higher among men than among women, and among white than among Afro-descendent individuals. Less than one-third of individuals with SO had 6-11 years’ education, while just more than one-third reported an income of >5 MW (Table 2).

The prevalence of SO among people with and without arthrosis/arthritis was significantly different, but was not different among those with and without DM, and was similar between those with arthrosis/arthritis and those with DM (*p*<0.0001) (Table 2).

Among the lifestyle variables, a high prevalence of SO was observed in older adults who had stopped smoking and who drank alcohol daily, but these findings lacked statistical significance. SO was also present in more than one-quarter of older adults who did not walk for exercise, but this was not statistically significant (Table 2).

Analysis of the biomarkers showed that about one-fifth of older adults with SO also had hypoalbuminemia, about one-third had hyperglycemia, and about one-quarter had elevated CRP levels (Table 2).

In terms of functional variables, among individuals with SO, more than 30% had lower gait speed, more than one-third had reduced hand-grip strength, and about one-third were dependent according to the IADLs (Table 2).

Table 3 shows the means and standard deviations for body composition characteristics, by presence of obesity and SO. Individuals with SO had higher mean fat percentage values than eutrophic individuals (*p*<0.001). The percentage of fat in obese individuals was exceeded when wishing with sarcopenic obsession. Individuals with SO also had lower mean muscular mass index values than obese individuals (*p*<0.001).

Table 4 states the crude and adjusted ORs for the associations between the independent variables (functional, morbidity, lifestyle, and biomarker) and SO. The crude analyses showed that lower gait speed, daily consumption of alcohol, and hyperglycemia displayed significant associations with SO. In the final model, adjusted by the demographic and socioeconomic variables, lower gait speed, arthrosis/arthritis, and hyperglycemia were significantly associated with SO.

In Table 5, the crude and adjusted ORs for the associations between the independent variables and obesity are stated. In the final model, adjusted by the demographic and socioeconomic variables, lower gait speed, arthrosis/arthritis, and hyperglycemia were significantly associated with obesity.

## DISCUSSION

This study found a high prevalence of SO and obesity among older adults in Brazil. These results corroborate other Brazilian and international studies (1,16,18,26,27,30). The prevalence of SO in Brazilian studies have ranged from 19.6% to 34.2% (30,32,39,40). Globally, studies of non-institutionalized older adults have reported an SO prevalence ranging from 2.1% to 94% (14,15,19,40,41). In Brazil, a cross-sectional study of 272 older women by Silva et al. (30) found a prevalence of SO of 34.2%, based on DXA evaluations (30). A cross-sectional study of 306 older adults in Spain found a prevalence of SO of 25% (10).

The present study found a higher prevalence of SO among men (65.3%) than among women (34.6%). This was a similar trend to that found by Batsis et al. (41), in a cross-sectional study in the United States by the National Health and Nutrition Examination Survey III—1999-2004. In a sub-sample of 4,984 non-institutionalized older adults of both sexes, aged 60 years or more, using cut-off points proposed by the Foundation for the National Institutes of Health, they observed a prevalence of SO, as evaluated by appendicular skeletal muscle mass, of 27.8% in men and 19.3% in women. However, contradictory results were observed in the study (19) using data from the National Health and Nutrition Examination Survey (1999-2004) for 4,984 older adults in the United States, using different definitions from six studies (4,5,42-45) and different diagnostic criteria for SO. In that study, they found widely differing prevalence values for SO, which were often higher among women, ranging from 3.6% to 94.0%, while among the men the values varied from 4.4% to 84.0%.

Thus, the prevalence of SO is not yet clearly established and can vary by a factor of as much as 26, mainly because of the use of different evaluation methods, criteria, and cut-off points applied to determine muscle mass and fat mass (19). This variability underlines the need to establish diagnostic criteria and cut-off points in order to build a consensus in the scientific literature (23). In addition, ethnic differences may influence the prevalence of SO, given that oriental populations have less muscle mass and fat mass than western populations (46).

Moon et al. (6), in a study of 1,583 older adults in Korea between 2008 and 2010, found a lower SO prevalence: 7.8% for men and 9.6% for women. Moreover, Kim et al. (26) analyzed baseline data for 493 older adults from the Korean Sarcopenic Obesity Study (KSOS) and found a prevalence of SO of 17.8% for men and 24.9% for women.

It has been suggested that obesity can lead to sarcopenia, and *vice versa* (46-48). The underlying mechanism is related to the production of substances in fatty tissue, such as tumor necrosis factor and leptin, which influence insulin resistance, reduction of energy metabolism, and secretion of growth hormone and proinflammatory cytokines, leading to progressive muscle mass loss and body fat increase, particularly by fatty infiltration of muscle (47). Muscle with high levels of infiltrated fat may be more inflamed than fat-free muscle, suggesting a connection between fat mass gain, and muscles with increased triglycerides and inflammation (47,49). Increased adiposity leads to chronic subclinical inflammation, which fosters muscle catabolism and can lead to sarcopenia (50).

Thus, the results of this study, which showed a high prevalence of obesity (44.2%) among non-institutionalized older adults, can serve as an alert to the greater risk of such older adults developing SO in the near future. The prevalence of SO and obesity is probably associated with the specific characteristics of our sample, which included older individuals and mostly women. Indeed, in other studies, the proportion of SO and obesity increased with age, most likely due to hormone-related body composition changes, reaching higher values in individuals over 75 years of age.

The results of this study confirm the associations of SO with gait speed, hyperglycemia, and arthrosis, as reported in international studies (5,51). Lower gait speed is an important indicator of functional impairment in older adults, and this study found that older adults with reduced gait speed were twice as likely to display SO. A study by Chang et al. (9), in Taiwan examined data for 2,629 older adults from the Sarcopenia and Translational Ageing Research in Taiwan (START) survey, to evaluate the association between functionality and body composition. They found an association between lower gait speed and SO (*p*<0.005). Moreira et al. (52), in a cross-sectional study of 491 participants in Parnamirim, Brazil, observed that variables relating to physical (functional) performance displayed statistically significant association with SO, although there was no statistical significance to the association between gait speed and SO. This vicious cycle of functional incapacity and slower gait leads to a reduction in overall energy expenditure, reduced muscle mass, and increased fat mass in older adults.

Our findings in relation to elevated glycaemia were in agreement with the findings of the study in Korea by Hwang et al. (51), which examined data from the Fourth Korea National Health and Nutrition Examination Survey (2009), a population survey of 2,221 older adults. They found that older adults with hyperglycemia had higher odds of developing SO. Santos et al. (1), in a cross-sectional study in Brazil, examined 149 older women and found a positive correlation between glucose levels and SO (*p*<0.05).

It remains unclear whether the presence of arthrosis/arthritis precedes SO or whether individuals with SO are at higher risk of developing arthritis/arthrosis. The possibility of bidirectionality between such events should not be discarded as it is quite plausible. However, we found no other study that has evaluated the role of arthrosis/arthritis in the development of SO. Tyrovolas et al. (8) analyzed data from the Collaborative Research on Ageing in Europe (COURAGE) Survey and the World Health Organization Study on Global Ageing (SAGE), which comprised 18,363 older adults from several different geographical regions of the world, between 2007 and 2012. They observed that older adults with a chronic disease were 1.8 times more likely to develop SO.

This study had some limitations. Its cross-sectional design did not enable temporal or causal associations to be shown, leading to the possibility of reverse causation among the findings. This possibility cannot be excluded, given the bidirectionality of the phenomena studied. Another limitation involved the use of walking as physical exercise to assess the physical activity of the participants. This assessment is insufficient to assess the set of physical activities that are performed, and thus may have led to underestimation of this parameter. Additionally, there may have been information bias resulting from the self-reported physical morbidity data. The scarcity of studies and the great variability of diagnostic methods and criteria for SO placed limitations on data comparability. Moreover, the FIBRA-RJ study involves a closed cohort. This fact prevented the sample from being recomposed after losses occurred during the follow-up and exclusions during data collection. Despite these limitations, the specificities of the FIBRA-RJ sample has the advantage in terms of providing knowledge about the body composition phenotypes present in the elderly population.

To the best of our knowledge, no previous study had evaluated the prevalence of SO and its associations with functionality, lifestyle, biomarkers, and physical morbidity in Brazil, using the DXA method. Data quality was assured by team training, data quality supervision and control immediately upon completion of the data collection protocols, and prompt correction of any errors. Lastly, evaluation of body composition by the DXA method, which is considered to be a reference method, provided greater precision and validity to the study findings.

## CONCLUSION

In summary, this study revealed a high prevalence of obesity and SO among older adults in Brazil. The high prevalence of both these two body composition phenotypes are cause for concern, because obese older adults can become sarcopenic obese. The finding that slower gait, hyperglycemia, and arthrosis are associated with the presence of SO underscores the possibility that early intervention and prevention of these modifiable risk factors can assist in preventing obesity and SO in older adults.

Despite limited data comparability due to cultural and racial differences, and diversity in cut-off points and diagnostic criteria, the results of various different studies reinforce the association between lower gait speed, hyperglycemia, and SO in older adults. To improve research in this field and ensure better data comparability, it is of prime importance that consensus on the diagnosis of SO be established.

## AUTHOR CONTRIBUTIONS

Campos GC contributed with significant participation in the study design, data collection, analysis, interpretation of data and preparation of the article. Lourenço RA contributed with significant participation in the study design, preparation or revision of the manuscript and approval of the final version of the manuscript for publication. Lopes CS contributed with significant participation in the study design, preparation and review of the analysis and manuscript and approval of the final version for publication.

## Figures and Tables

**Table 1:** Frequency of socioeconomic and demographic characteristics, morbidities, lifestyle, biomarkers, body composition, and functional phenotypes (FIBRA-RJ-2013).

Characteristics		N (270)	(%)
Sex	Male	81	30.0
Ethnicity	Afro-descendent	99	36.7
Income bracket (*MW)	0-2	49	19.1
	2.1-5	119	46.5
	>5	88	34.4
Age group (years)	65-74	101	37.4
	75-84	127	47.0
	≥85	42	15.6
Schooling (years)	Illiterate	1	0.4
	1-5	53	19.6
	6-11	125	46.3
	>12	91	33.7
Diabetes mellitus	Yes	68	25.3
Arthrosis/Arthritis	Yes	133	49.3
Tobacco use	Current smoker	12	4.4
	Smoked and stopped	97	35.9
	Never smoked	161	59.6
Alcohol consumption (daily)	Yes	37	13.7
Walking for exercise	Yes	64	23.7
Albumin (<3.5 mg/dl)	Deficit	22	8.2
C-reactive protein (≥0.5 mg/dl)	High	43	16.0
Glycaemia (≥100 mg/dl)	High	121	45.2
Body composition phenotypes	Eutrophic	70	26.4
	Obese	117	44.2
	Sarcopenic Obese	78	29.4
IADL**	Dependent	97	35.9
Gait speed	≤0.8m/s	118	43.9
Hand grip	Low	69	26.3

* MW—Minimum Wages;

** IADL—Instrumental Activities of Daily Living.

**Table 2:** Prevalence of sarcopenic obesity, by sociodemographic and economic characteristics, lifestyle, morbidities, and biochemical and functional markers (FIBRA-RJ-2013).

Variables		Eutrophy		Obesity		Sarcopenic obesity		*p*-value
		N	(%)	N	(%)	N	(%)	
Sex	Female	46	25	111	60.3	27	14.7	0.0001
	Male	24	29.60	6	7.4	51	63	
Ethnicity	White	39	23.35	69	41.3	59	35.33	0.021
	Afro-descendent	31	31.63	48	48.98	19	19.39	
Income bracket (*MW)	0-2	8	16.67	22	45.83	18	37.50	0.010
	2.1-5	30	25.86	60	51.72	26	22.41	
	>5	29	33.33	26	29.89	32	36.78	
Age group (years)	65-74	23	23.00	50	50.00	27	27.00	0.609
	75-84	34	27.42	52	41.94	38	30.65	
	≥85	13	31.71	15	36.59	13	31.71	
Schooling (years)	Illiterate	0	0.00	1	100.00	0	0.00	0.254
	1-5	14	28.00	26	52.00	10	20.00	
	6-11	29	23.39	59	47.58	36	29.03	
	≥12	27	30.00	31	34.44	32	35.56	
Diabetes Mellitus	Yes	17	25.00	36	52.94	15	22.06	0.202
	No	53	27.04	81	41.33	62	31.63	
Arthrosis/Arthritis	Yes	26	19.7	76	57.6	30	22.7	0.000
	No	44	33.1	41	30.8	48	36.1	
Tobacco use	Yes	4	33.33	4	33.33	4	33.33	0.020
	No	40	25.3	82	51.9	36	22.8	
	Smoked and stopped	26	27.4	31	32.6	38	40.0	
Daily consumption of alcohol	Yes	7	18.92	12	32.43	18	48.65	0.022
	No	63	27.63	105	46.05	60	26.32	
Walking for exercise	Yes	19	30.16	19	30.16	25	39.68	0.029
	No	51	25.25	98	48.51	53	26.24	
Albumin	Normal	69	28.5	101	41.7	72	29.8	0.007
	Deficit	1	4.8	16	76.2	4	19.0	
C-reactive protein	Normal	63	28.6	91	41.4	66	30.0	0.061
	High	7	16.3	26	60.5	10	23.3	
Glycemia	Normal	48	33.8	57	40.1	37	26.1	0.017
	High	22	18.2	60	49.6	39	32.2	
Gait speed	≤0.8m/s	20	17.4	62	53.9	33	28.7	0.004
	>0.8m/s	50	33.6	54	36.2	45	30.2	
Hand grip	Low	15	21.7	29	42	25	36.2	0.320
	Normal	55	28.1	88	44.9	53	27.0	
IADL**	Independent	47	27.6	76	44.7	47	27.6	0.820
	Dependent	23	24.2	41	43.2	31	32.6	

* MW—Minimum Wage;

** IADL—Instrumental Activities of Daily Living.

**Table 3:** Measures and standard deviation of body composition characteristics, by body composition phenotypes and anthropometry (FIBRA-RJ-2013).

Body Composition and Anthropometry	Obesity			Sarcopenic Obesity			Total			
	N	Mean	SD	N	Mean	SD	N	Mean	SD	*p*-value
Fat Percentage (%)	117	44.9	3.7	78	35.6	4.5	265	38.5	7.5	<0.001
ASMMI (Kg/m^2^)*	117	7.5	1.7	78	5.1	0.8	265	5.9	2.0	<0.001
Weight (Kg)	117	73.6	12.5	78	67.4	12.6	265	68.0	13.4	<0.001
Waist Circumference (cm)	117	102.5	10.5	78	96.8	8.9	265	96.6	12.0	<0.001

* ASMMI—Appendicular Skeletal Muscle Mass Index.

**Table 4:** Crude and adjusted odds ratios (OR) and respective 95% confidence intervals (95%CI) for the association between sarcopenic obesity and functional variables and morbidities, lifestyle and biomarkers (FIBRA-RJ -2013).

	Sarcopenic Obesity				
		Model 1	Model 2	Model 3	Final Model
Variables	OR (95%CI)	Adjusted OR (95%CI)	Adjusted OR (95%CI)	Adjusted OR (95%CI)	Adjusted OR (95%CI)
Gait speed					
>0.8 m/s	1	1			1
≤0.8 m/s	1.83 (0.92-3.64)	2.01 (0.94-4.32)			2.43 (1.09-5.41)
Arthrosis					
No	1	1			1
Yes	1.05 (0.54-2.05)	2.05 (0.92-4.53)			2.43 (1.05-5.63)
Diabetes mellitus					
No	1	1			1
Yes	0.75 (0.34-1.65)	0.69 (0.29-1.67)			0.52 (0.19-1.37)
Smoking					
Never smoked	1		1		1
Smoker	1.11 (0.25-4.77)		0.67 (0.13-3.51)		0.23 (0.03-1.75)
Smoked and stopped	1.62 (0.82-3.18)		0.94 (0.43-2.08)		0.75 (0.31-1.76)
Daily drinker					
No	1		1		1
Yes	2.70 (1.05-6.92)		2.29 (0.81-6.46)		2.63 (0.84-8.20)
Walks					
Yes	1		1		1
No	0.79 (0.38-1.60)		0.94 (0.42-2.09)		0.86 (0.36-2.02)
Albumin					
Normal	1			1	1
Deficit	3.83 (0.41-35.15)			7.24 (0.72-72.85)	5.70 (0.54-59.89)
C-Reactive Protein					
Normal	1			1	1
High	1.36 (0.48-3.80)			1.38 (0.45-4.19)	1.22 (0.37-4.02)
Glycemia					
Normal	1			1	1
High	2.30 (1.17-4.52)			2.15 (1.02-4.55)	3.14 (1.34-7.32)

Model 1**—**Block of functional variables and morbidities (gait speed, arthrosis and diabetes mellitus)+adjusted for sociodemographic and economic variables (sex, income bracket and ethnicity).

Model 2**—**Block of lifestyle variables (tobacco use, daily consumption of alcohol and walking for exercise )+adjusted for sociodemographic and economic variables.

Model 3**—**Block of biochemical marker variables (glycaemia, albumin and C-reactive protein)+adjusted for sociodemographic and economic variables.

Model 4**—**Final; Functional variables and morbidities, lifestyle, biochemical markers+adjusted for sociodemographic and economic variables.

**Table 5:** Crude and adjusted odds ratios (OR) and respective 95% confidence intervals (95%CI) for the association between obesity and functional variables and morbidities, lifestyle and biomarkers (FIBRA-RJ -2013).

		Obesity			
		Model 1	Model 2	Model 3	Final Model
Variables	OR (95%CI)	Adjusted OR (95%CI)	Adjusted OR (95%CI)	Adjusted OR (95%CI)	Adjusted OR (95%CI)
Gait speed					
>0.8 m/s	1	1			1
≤0.8 m/s	2.87 (1.52-5.44)	2.01 (0.94-4.32)			3.12 (1.45-6.67)
Arthrosis					
No	1	1			1
Yes	3.13 (1.69-5.80)	2.05 (0.92-4.53)			2.54 (1.20-5.39)
Diabetes mellitus					
No	1	1			1
Yes	1.38 (0.70-2.71)	0.69 (0.29-1.67)			1.09 (0.42-2.81)
Smoking					
Never smoked	1		1		1
Smoker	0.48 (0.11-2.05)		0.40 (0.08-1.86)		0.33 (0.06-1.73)
Smoked and stopped	0.58 (0.30-1.10)		0.90 (0.42-1.91)		0.80 (0.35-1.83)
Daily drinker					
No	1		1		1
Yes	1.02 (0.38-2.74)		2.36 (0.75-7.43)		2.98 (0.89-9.97)
Walks					
Yes	1		1		1
No	1.92 (0.93-3.94)		1.36 (0.60-3.10)		1.16 (0.48-2.78)
Albumin					
Normal	1			1	1
Deficit	10.93 (1.41-84.34)			8.55 (1.05-69.35)	6.23 (0.72-53.74)
C-Reactive Protein					
Normal	1			1	1
High	2.57 (1.05-6.28)			1.71 (0.64-4.58)	1.76 (0.63-4.93)
Glycemia					
Normal	1			1	1
High	2.29 (1.23-4.27)			2.70 (1.32-5.55)	3.09 (1.33-7.14)

Model 1**—**Block of functional variables and morbidities (gait speed, arthrosis and diabetes mellitus)+adjusted for sociodemographic and economic variables (sex, income bracket and ethnicity).

Model 2**—**Block of lifestyle variables (tobacco use, daily consumption of alcohol and walking for exercise )+adjusted for sociodemographic and economic variables.

Model 3**—**Block of biochemical marker variables (glycaemia, albumin and C-reactive protein)+adjusted for sociodemographic and economic variables.

Model 4**—**Final; Functional variables and morbidities, lifestyle, biochemical markers+adjusted for sociodemographic and economic variables.
